# Medical News and Miscellany

**Published:** 1896-07

**Authors:** 


### Medical News and Miscellany.

**A small announcement** in our May number, that a sober physician could find employment at a certain small station in Harris county brought nineteen inquiries, some from well known physicians. Among them were two which we copy below, as showing that the ignorant and uneducated are not alone amongst the quacks. These men have diplomas from reputable colleges, and one of them is in affiliation with the Texas State Medical Association. Names, of course, are suppressed. How the college got over that clause requiring as conditions to matriculation, "evidences of a good English education," is a mystery. We need something more than a bill to regulate the practice. We need a higher standard of education on the part of those who are given diplomas. The attention of the Medical College Association is called to the fact that men who can not write the English language correctly are matriculated and allowed to graduate:

———, Tex
June 5th, '96

—— —— —— ——

Dear sir I notic in Danels Tex Medical Journal that a physician is wanted at ——, hence I rite the ask in regard to the location and what the population of the place and surounding country has, and do you think the it will support a Dr, and give me any othe information you have

I am Respectfuly, —. —. ———, M D.

———

——, co
Tex

———

——— tex
may 21–96

—— —— ——

———

Harris Co tex
Dear Sur I notice in the texas medical Journel that ther is wanted a Dr of medicin at ——— tex on the m. K. x T. R R I want a Location of that Kind
and would Like to Have all of the Details of the Place and Country Round there. Will Be Pleased to Hear From you Soon yours
Respectfulley
—. —. ——— m D.
——, —— Co tex
P. S. I wille Give Referance if Desired

**The Medical Department** of the University of Texas, at Galveston, contains the following notable nest of graduates of the Medical School (University of Pennsylvania): Allen J. Smith, '86, Professor of Physiology and Clinical Lecturer on Mental and Nervous Diseases; Edward Randall, '83, Professor of Therapeutics and Materia Medica; David Cerna, '79, Demonstrator of Physiology and Lecturer on the History of Medicine. Dr. J. F. Y. Paine, Professor of Obstetrics, took his first year at our Medical School, but withdrew at the opening of the civil war, and received his degree from what is now Tulane University, La. Professor Smith and Dr. Cerna are connected with the editorial staff of the TEXAS MEDICAL JOURNAL, the most prominent medical journal in that State, published at Austin.—*Alumni Register*.

### A Warning to Druggists.

NEW YORK, June 12, 1896.

*Editor Texas Medical Journal:*

DEAR SIR:—A paragraph is going the rounds of the medical journals, giving a formula for making palatable castor oil.

This formula is patented by a long list of patents [list omitted], and if druggists are induced to prepare this article themselves, it will lead to a multitude of law suits like those instituted in the "Drive Well" case.

Some scheming lawyer would like to take up this case for one-half the profits, and I think journals should warn the druggists so that they may not be caught in a trap.

Yours truly,
A. J. WHITE.

**Ownership of the Prescription.**—The question has been asked us what has been the decision of the courts as to the ownership of a prescription, and not recalling any decision on the subject, we sought information of Mr. Carleton, President of the Pharmacy Board. He says that the decision in several States has been that the property of the prescription resides in the patient; the one who paid for it and for whom it was written. In Texas, there has been no decision of any court, so far as his knowledge extends. We will thank any one of our readers to cite us to any decision on the subject, should any of them be in possession of the information,—if there has been any decision in this State.

**Dr. R. M. Swearingen,** State Health Officer of Texas, an A. M. of Centenary College, Louisiana, by invitation, delivered the annual address before the Literary Societies of the Uuiversity of Texas, at the commencement, held at the close of the session of 1895 and '96, June 14th to 18th. His subject was "Life: The Indestructability of Matter a Basis for Belief in a Future State." A large and appreciative audience assembled to hear the noted orator, and for an hour gave him a breathless attention. It was a splendid effort, and worthy of his reputation. Delivered *ex tempore*, his oratory was grand,—and some thoughts, glowingly expressed, almost touched the sublime.

**Dr. Sam R. Burroughs'** correspondents will please take notice of his change of address from Raymond, Leon county, to Buffalo, Leon county. See his advertisement in this issue of place to sell at a big bargain.

**The Texas Medical Windmill.**—The *Texas Medical News* says Dr. Daniel is fighting a windmill. As the *Texas Medical News* is the only thing we have been fighting, having knocked the wind out of the mixed-board-bill, we suppose that publication was intended as referred to; an apt designation.

---

**In a private** letter to the editor, Dr. Bibb, of the City of Mexico, extols oxygen inhalations in the treatment of typhus fever, pneumonia, and in the graver forms of malarial fevers as a tonic, a stimulant and as a powerful antiseptic in these diseases.

---

**Hymeneal.**—On 20th of June (ult.), Dr. R. L. Miller was married to Miss Lena May York, daughter of Dr. W. L. York, all of Decatur, Texas. The JOURNAL extends its congratulations.

---

**Dr. J. T. Musick** has moved from Pittsburg, Texas, to Quintana, Texas.

---

**Dr. S. H. Stout,** now of Dallas, the well known Medical Director of Hospitals of the late Confederate States, has been appointed Assistant Surgeon-General on the staff of General Gordon, Commander-in-Chief of the Association of United Confederate Veterans, with the rank of general. Dr. Stout succeeds Dr. C. H. Thebault, who was promoted to the position of Surgeon-General upon the death of Surgeon-General Joseph Jones. Many of Dr. Stout's friends thought he should have been Surgeon-General, but as the organization is historical, benevolent and social, considering the doctor's age, and his remoteness from headquarters, as well as Dr. Thebault's familiarity with the position, it is eminently more fitting that it should be as it is. Dr. Stout takes that view of it. We extend to the doctor our congratulations.

---

**Location Exchange.**—Every physician who is seeking a location in Texas, is requested to communicate with the JOURNAL, stating what he wants; and every physician who wants to sell out and move, is requested also to write us, giving full information of what he has to sell. We will endeavor to effect sales and secure locations, charging a small agent's fee where a trade is made. We have a list now of some practices for sale, and there are several prospective buyers corresponding with us.

**"The Doctor in Politics."**—Dr. W. R. Hayden, of Bedford Springs, Mass., the venerable physician and President of the New York Pharmaceutical Co., has been elected to the Massachusetts Legislature—standing in the House of Representatives—for the historic towns of Concord, Lexington, Lincoln, Burlington and Bedford—a most distinguished constituency. He is the oldest man in the assembly and is recognized as the "Father of the House." We are pleased to learn that the Doctor's health is very good, and we hope that he will be spared for many more years of usefulness. He is an honor to the Legislature. The Doctor renews the advertisement of the celebrated Hayden's Viburnum Compound for the twelfth consecutive year in the "Red Back." See it.

---

**The Doctor to the Front.**—It is said that Dr. A. W. Fly, the up-to-date mayor of Galveston, is likely to be the choice of the gold wing of the Democratic party for governor, when they hold their nominating convention in Waco, August 17, prox. Dr. Fly is a vigorous man, a man of great versatility of talent. He would make a good anything where sense and strength of character, i. e., backbone—are required. As mayor of Galveston he has given evidence of both administrative and executive talent of high order. As governor, we are sure he would make a record, and a rattling good one.

---

**The** JOURNAL will be pleased to have the physicians of Texas give their views, through its columns, upon the subject discussed in this issue. Our Austin contemporary says Dr. Daniel is playing *solitaire*. We do not believe we are alone in objecting to our State Association asking for a mixed board, and we ask for an expression on the subject, by all who feel sufficiently interested to drop us a line.

---

**Von Boeckmann** gets the Transactions. He will print 550 copies, in paper binding, for $363, or 66 cents per copy. Told you so. This will save the Association something like $200. Dr. Daniel's "criticisms" have taken effect at last, and to some purpose.

---

**A Rare Opportunity** is offered a competent and energetic physician to secure one of the best locations to practice to be found anywhere. Dr. Sam R. Burroughs, who has held the practice of the larger part of East Leon County for twentyfive years, has been induced t[illegible] go to the railroad (Buffalo) for convenience of patients, and offers for less than cost of residence, his home, consisting of a well improved farm of 206 acres, with growing crops, seven-room residence nearly new, out houses, bath house, milk house, stables, three tenant houses, fine orchard, his office and equipments—all for $1500. His practice and good will go to the purchaser. Churches and schools; healthy home beautifully improved. Here is a rare chance to secure a lovely home and paying practice for a small consideration. Address Dr. Burroughs, at Buffalo, Leon Co., Tex., or this journal.

---

**National Conventions.**—The International and Great Northern railway will sell round trip tickets at one fare rate for the following national conventions:

Democratic—Chicago, July 7.

Populist—St. Louis, July 22.

Call on agent for full particulars.

D. J. Price, A. G. P. A.,
Galveston, Texas.

